# Poor CD4 recovery and risk of subsequent progression to AIDS or death despite viral suppression in a South African cohort

**DOI:** 10.7448/IAS.17.1.18651

**Published:** 2014-03-03

**Authors:** Simbarashe Takuva, Mhairi Maskew, Alana T Brennan, Lawrence Long, Ian Sanne, Matthew P Fox

**Affiliations:** 1 Clinical HIV Research Unit, Department of Internal Medicine, School of Clinical Medicine, Faculty of Health Sciences, University of the Witwatersrand, Johannesburg, South Africa; 2 Health Economics and Epidemiology Research Office, Department of Internal Medicine, School of Clinical Medicine, Faculty of Health Sciences, University of the Witwatersrand, Johannesburg, South Africa; 3 Center for Global Health and Development, Boston University, Boston, MA, USA; 4 Department of Internal Medicine, School of Clinical Medicine, Faculty of Health Sciences, University of the Witwatersrand, Johannesburg, South Africa; 5 Right to Care, Johannesburg, South Africa; 6 Department of Epidemiology, Boston University School of Public Health, Boston, MA, USA

**Keywords:** discordance, immunologic response, CD4 cell count, viral suppression, AIDS, disease progression, antiretroviral therapy

## Abstract

**Introduction:**

The prognostic role of CD4 response in the first six months of treatment in patients achieving early viral suppression during HIV treatment is unclear.

**Methods:**

This was a cohort study of HIV-positive adults initiating antiretroviral therapy (ART) between April 2004 and August 2007 who achieved viral suppression (<400 copies/ml) by six months on treatment in South Africa. Immunological response at six months was defined as: (1) absolute CD4 reached (<200 vs. ≥200 cells/ml); (2) absolute CD4 reached (0–49, 50–200 and ≥200 cells/ml); and (3) CD4 increase from ART initiation (<0, 0–49, 50–199 and ≥200 cells/ml). We used Cox regression models to determine the relationship between each definition and both new AIDS-defining condition and death.

**Results:**

A total of 4129 patients were eligible for analysis; 212 (5.1%) of those patients experienced a new AIDS-defining condition and 154 (3.7%) died. Smaller CD4 gains by six months were associated with higher hazards of progression to AIDS (CD4<50 vs. ≥200 cells/ml; adjusted hazard ratio (aHR): 2.6; 95% CI: 1.2–2.1) and death (aHR: 2.8; 95% CI: 1.4–5.7). A decrease in CD4 count since ART initiation through six months (aHR: 2.4; 95% CI: 1.2–4.9) and smaller CD4 count gains (0–49 cells/ml; aHR: 2.0; 95% CI: 1.2–3.4 and 50–199 cells/ml; aHR: 1.5; 95% CI: 0.9–2.2) were also associated with greater risk of progression to AIDS compared to an increase of ≥200 cells/ml. When we examined mortality differences by gender among this virally suppressed cohort, a higher proportion of males died compared to females, 4.7% versus 3.2%, *p=*0.01. However, in multivariable analysis, we did not observe any significant differences: aHR: 1.39; 95% CI: 0.98–1.95.

**Conclusions:**

Patients on ART with poor CD4 recovery early in treatment are at greater risk of progression to new AIDS diagnosis or death despite viral suppression. Approaches to managing this sub-group of patients need further investigation.

## Introduction

In HIV-positive patients, antiretroviral therapy (ART) has been demonstrated to strongly reduce both morbidity and mortality associated with HIV infection through suppression of HIV viral load and restoration of immune function [1–4]. CD4 count and viral load are both strong predictors of disease progression and survival regardless of whether a patient is on treatment [5–12]. Although these two factors are strongly interrelated, they are not perfectly correlated. Even among those who achieve full viral suppression, between 7% and 40% of patients still show poor CD4 recovery, leaving them at significant increased risk of adverse clinical outcomes [13–27].

Studies investigating the association of poor CD4 recovery during viral suppression and adverse clinical outcomes amongst patients on ART have shown conflicting results [14,15,22,27–33]. Varying definitions of CD4 count recovery have been employed across studies, mainly because no uniform definition of immunologic success on ART exists, making it difficult for clinicians to synthesize the results and draw conclusions [14,15,16,17,19,24,27,33]. A majority of studies show evidence for an increased risk for progression to AIDS or death among those with poor immune recovery while fewer studies show little or no effect. Commonly, an absolute CD4 count increase of ≥50 cells/ml at either 6- or 12-months post-ART initiation has been employed to define appropriate CD4 count recovery [16,17,19], yet several studies have used CD4 count increases of 100 cells/ml at 6- or 12-months [14,33]. Percentage increase in CD4 count (i.e. ≥30% increase from baseline value) and absolute values achieved at six months (CD4 count ≥200 cells/ml) have also been used [15,24,27]. These varying definitions have obscured our ability to clearly measure and summarize this important relationship.

To understand the impact of CD4 response despite virologic suppression on clinical outcomes, we studied the impact of three definitions of immunological response by six months post-ART initiation amongst patients that achieved viral suppression by six months on treatment in a large cohort in Johannesburg, South Africa. These three definitions focused on: (1) an absolute CD4 count level achieved; (2) categories of CD4 count achieved; and (3) change in CD4 count from ART initiation.

## Methods

### Study population

The Themba Lethu HIV Clinic in Johannesburg, South Africa is one of the largest HIV care and treatment clinics in South Africa with over 21,000 patients initiated onto ART since 2004. Details of this cohort have been published previously [34,35].

### Study cohort eligibility criteria

We conducted a prospective cohort analysis of HIV-positive ART naïve patients ≥18 years of age, who initiated standard first-line ART at Themba Lethu between April 2004 and August 2007 with CD4 counts ≤350 cells/ml. At the time, standard public-sector ART in South Africa consisted of stavudine (d4T) or zidovudine (AZT) with lamivudine (3TC) and either efavirenz (EFV) or nevirapine (NVP). All patients initiating ART with CD4 count less than 200cells/ml were eligible to receive cotrimoxazole prophylaxis [36]. However, we did not have cotrimoxazole uptake information in the database. Given that this study was to explore impact of CD4 responses on the clinical outcomes of patients who had completed at least six months of ART and further had a suppressed viral load, we limited the analysis to patients who completed six months of treatment and achieved viral load suppression (<400 copies/ml) in that time. Patients either dying, lost to follow-up or not suppressing viral load within the first six months of ART initiation were thus excluded. Patients missing CD4 counts at both ART initiation (defined as six months before to seven days after ART initiation) and six months on treatment (defined as between four and eight months) were further excluded from the analyses. During the study period CD4 counts and viral loads were done between four and six months after ART initiation and then six monthly thereafter [36].

### Variable definitions

We used three specifications of CD4 response at six months post-ART initiation: (1) achieving a CD4 count >200 cells/ml at six months post-ART initiation; (2) CD4 count further categorized (0–49, 50–200 and ≥200 cells/ml) at six months post-ART initiation; and (3) change in CD4 at six months from the ART initiation value (<0, 0–49, 50–99 and ≥200 cells/ml). We explored the impact of the varying levels of immunologic response in virally suppressed patients on two outcomes that occurred six months post-ART initiation: progression to a new AIDS-defining condition and death. The revised 2008 US Centers for Disease Control and Prevention AIDS case definition was used to define a new AIDS condition [37]. At Themba Lethu, mortality is ascertained via family or hospital report, active tracing and linkage with the South African National Vital Registration Infrastructure Initiative, a system estimated to have 90% sensitivity for adults [38,39].

For new-AIDS condition and death, person-time accrued from six months after treatment initiation until the earliest of: (1) new AIDS-defining condition; (2) death; (3) lost to follow-up (defined as four months late for last scheduled clinic visit); (4) transfer; or (5) completion of 24 months of follow-up. For all patients, time zero was the date of viral load suppression (approximately six months after ART initiation). If patients had new-AIDS condition or death, they stopped contributing person-time at the time-point when the new AIDS condition started.

### Statistical analysis

Patient baseline characteristics were summarized with descriptive statistics. Incidence rates (IR) per 1000 person-years (PY) were computed between 0–6, 6–12, 12–18 and 18–24 months after viral suppression (at six months) to compare the occurrence of study outcomes over follow-up. Kaplan–Meier curves were used to compare progression to AIDS or death stratified by each definitions of immunologic response. We estimated crude and adjusted hazard ratio (aHR) of progression to a new AIDS-defining condition and death using Cox proportional hazards models. Covariates that could plausibly confound the relation between CD4 response and the end-points that had a *p*<0.20 in bivariate analyses were included in the multivariate models. All multivariate models were adjusted for age, gender, pregnancy, haemoglobin (Hb) and body mass index (BMI) by six months post-ART initiation. For patients with missing BMI and Hb values at six months, the ART initiation value was carried forward. Person-time accrued from six months after treatment initiation hence this time-point was the study “baseline.” This was to avoid immortal time bias given that the exposure is defined at six months post-ART initiation hence the outcomes under study could not have occurred before then. In additional analyses, we further used Cox proportional hazards models to examine the hazard of progression to new AIDS condition and death after viral suppression by gender. The proportionality assumption was checked (using log (-log (survival)) over time for each covariate) and was not violated. Data were analyzed using STATA version 11 (Stata Corp., College Station, TX, US).

Use of Themba Lethu Clinic data was approved by the Human Research Ethics Committee of the University of the Witwatersrand and approval for analysis of anonymized data was approved by the Boston University Institutional Review Board.

## Results

### Baseline and follow-up characteristics

Between April 2004 and August 2007, 4803 ART naïve adult patients initiated standard first-line therapy at Themba Lethu and remained in care for at least six months. Of these, 4473 (93.1%) achieved viral suppression. Among the 4473 patients, 344 (7.7%) did not have CD4 count measures at both ART initiation and six months, leaving 4129 patients eligible for analysis. Patients excluded were similar in baseline characteristics, with the exception that those included were more likely to be unemployed (43.1% vs. 33.4%) and had higher Hb levels at ART initiation (median 11.6 g/d; Inter-quartile range (IQR) 10.1–13 vs. 11.1g/dl; IQR 9.4–12.7). Of those included, the majority were female (64.2%), with a median age of 40 years (IQR: 35–46) and on standard first-line regimen d4T-3TC-EFV (89.2%). The cohort was immunosuppressed at initiation of ART (median CD4 count 80 cells/ml; IQR: 29–146 increasing to 201; IQR: 130–287 cells/ml by six months post-ART initiation). Table 1 below shows characteristics at initiation of ART and six months post-ART initiation disaggregated by gender.

**Table 1:** Characteristics at initiation and at viral suppression among 4129 patients initiating ART at the Themba Lethu Clinic in Johannesburg, South Africa

Characteristics		Total	Males	Females
Characteristics at ART initiation				
Total		4129	1478 (35.8%)	2651 (64.2%)
Black race, *n* (%)		3939 (95.4%)	1401 (94.8%)	2538 (95.7%)
Employed, *n* (%)		1783 (43.2%)	730 (49.4%)	1053 (39.7%)
Hb	<10 g/dl, *n* (%)	983 (23.8%)	245 (17.0%)	738 (28.7%)
	≥10 g/dl, *n* (%)	3030 (73.4%)	1196 (83.0%)	1834 (71.3%)
CD4	<50 cells/ml, *n* (%)	1490 (36.1%)	630 (42.6%)	860 (32.4%)
	50–199 cells/ml, *n* (%)	2337 (56.6%)	758 (51.3%)	1579 (59.6%)
	≥200 cells/ml, *n* (%)	302 (7.3%)	90 (6.1%)	212 (8.0%)
BMI	<18.5 kg/m^2^, *n* (%)	654 (18.9%)	307 (24.6%)	347 (15.7%)
	≥18.5 kg/m^2^, *n* (%)	2807 (81.1%)	941 (75.4%)	1866 (84.3%)
Age	18–35 years	278 (6.7%)	138 (9.3%)	140 (5.3%)
	36–55 years	2845 (68.9%)	1109 (75.0%)	1736 (65.4%)
	>55 years	1006 (24.4%)	231 (15.6%)	775 (29.2%)
WHO	Stage I/II, *n* (%)	2173 (52.6%)	702 (47.5%)	1471 (55.5%)
	Stage III/IV, *n* (%)	1956 (47.4%)	776 (52.5%)	1180 (44.5%)
Regimen	EFV-3TC-d4T, *n* (%)	3681 (89.2%)	1368 (92.2%)	2318 (87.4%)
	EFV-3TC-AZT, *n* (%)	340 (8.2%)	72 (4.9%)	268 (10.1%)
	NVP-3TC-d4T, *n* (%)	98 (2.4%)	42 (2.8%)	56 (2.1%)
	NVP-3TC-AZT, *n* (%)	10 (0.2%)	1 (0.1%)	9 (0.3%)
Age (years), median (IQR)		39 (34–46)	41.1 (36.8–47.0)	39.0 (34.2–45.0)
Hb g/dl, median (IQR)		11.6 (10.1–13)	12.4 (10.7–14.0)	11.2 (9.8–12.6)
CD4 count, median (IQR)		80 (29–146)	69 (21–136)	87 (35–152)
BMI kg/m^2^, median (IQR)		21.5 (19.1–24.5)	20.3 (18.5–22.6)	22.4 (19.6–25.5)
Characteristics at six months post-ART initiation				
Hb	<10 g/dl, *n* (%)	615 (15.1%)	129 (8.9%)	486 (18.6%)
	≥10 g/dl, *n* (%)	3446 (84.9%)	1325 (91.1%)	2121 (81.4%)
CD4	<50 cells/ ml, *n* (%)	130 (3.2%)	79 (5.4%)	51 (2.0%)
	50–199 cells/ ml, *n* (%)	1888 (45.7%)	756 (51.2%)	1132 (42.7%)
	≥200 cells/ml, *n* (%)	2111 (51.1%)	643 (43.5%)	1468 (55.4%)
BMI	<18.5 kg/m^2^, *n* (%)	654 (18.9%)	307 (24.6%)	347 (15.7%)
	≥18.5 kg/m^2^, *n* (%)	2807 (81.1%)	941 (75.4%)	1886 (84.3%)
Hb g/dl, median (IQR)		12.1 (10.3–14)	12.6 (10.7–14.3)	11.9 (10.2–13.8)
CD4 count, median (IQR)		202 (133–286)	181 (113–262)	216 (146–298)
BMI kg/m^2^, median (IQR)		23.7 (21.1–27)	22.1 (20.3–24.7)	24.8 (21.9–28.3)

ART, antiretroviral therapy; Hb, haemoglobin; BMI, body mass index; EFV, efavirenz; 3TC, lamivudine; d4T, stavudine; AZT, zidovudine; NVP, nevirapine; IQR, inter-quartile range.

Over a median follow-up time of 23 months (IQR: 21.5–23.5) per person and 7029 total PY of follow-up, 212 (5.1%) patients experienced a new AIDS-defining condition, 154 (3.7%) died and 555 (13.4%) were lost to follow-up. For the CD4 count categories at initiation, the proportion lost to follow-up decreased roughly monotonically from 14.6% among those with 0–49 cells/ml to 14.0% among those 50–199 cells/ml, and for those with a CD4 count at ART initiation ≥200 cells/ml, 12.3% were lost.

Among 212 new AIDS-defining conditions, the most common were pulmonary tuberculosis (*n=*106; 50%), then extra-pulmonary tuberculosis (*n=*38; 17.9%) and *Mycobacterium avium* complex (*n=*3; 1.4%). Patients also developed oesophageal candidiasis (*n=*25, 11.8%), extra-pulmonary cryptococcosis (*n=*8, 3.8%), invasive cervical cancer (*n=*6, 2.8%), disseminated mycosis (*n=*6, 2.8%), Kaposi Sarcoma (*n=*6, 2.8%), non-Hodgkin's lymphoma (*n=*4, 1.9%), candidiasis of lungs (*n=*3, 1.4%), *herpes simplex* pneumonitis or oesophagitis (*n=*3, 1.4%), *pneumocystis jirovecii* pneumonia (*n=*3, 1.4%) and AIDS Dementia Complex (*n=*1, 0.5%). Table 2 shows the majority of new AIDS conditions occurred within the first zero to six months beyond viral suppression (i.e. 6–12 months on ART) [IR per 1000 PY 3.7; 95% CI: 3.0–4.5] and decreased with increasing time on ART (between 6 and 12 months post suppression 2.5/1000 PY; 95% CI: 1.9–3.3 and between 18 and 24 months post suppression 1.7/1000 PY, 95% CI: 1.2–2.5).

**Table 2:** Risk of progressing to new AIDS condition or death by different specifications of CD4 count recovery at viral suppression among the 4129 patients initiating ART at the Themba Lethu Clinic in Johannesburg, South Africa

	New AIDS condition		Death	
		
	Crude HR (95% CI) *p*	Adjusted HR (95% CI) *p*	Crude HR (95% CI) *p*	Adjusted HR (95% CI) *p*
Absolute CD4 count at six months				
<200 cells/ml	1.67 (1.27–2.21) <0.001	1.61 (1.21–2.15) 0.001	2.00 (1.44–2.79) <0.001	1.84 (1.30–2.60) <0.001
≥200 cells/ml	1.00	1.00	1.00	1.00
CD4 count category at six months				
<50 cells/ml	2.94 (1.64–5.27) <0.001	2.58 (1.16–2.08) 0.003	3.89 (2.03–7.44) <0.001	2.78 (1.35–5.73) 0.006
50–200 cells/ml	1.60 (1.64–5.27) <0.001	1.56 (1.39–4.79) 0.003	1.89 (1.35–2.65) <0.001	1.79 (1.26–2.55) 0.001
≥200 cells/ml	1.00	1.00	1.00	1.00
Change from the baseline value				
<0 cells/ml	2.23 (1.13–4.40) 0.021	2.43 (1.22–4.86) 0.012	2.65 (1.26–5.49) 0.009	2.00 (0.91–4.42) 0.090
0–49 cells/ml	2.02 (1.24–3.27) 0.004	2.04 (1.23–3.38) 0.006	1.74 (1.00–3.06) 0.054	1.48 (0.82–2.68) 0.192
50–199 cells/ml	1.44 (0.95–2.18) 0.083	1.46 (0.94–2.23) 0.091	1.33 (0.83–2.14) 0.234	1.29 (0.79–2.09) 0.310
≥200cells/ml	1.00	1.00	1.00	1.00

HR estimated using Cox proportional hazards regression models, adjusted for age, gender, haemoglobin (Hb), body mass index (BMI) and pregnancy status at six months post-ART initiation.

The association of CD4 count recovery with mortality showed a similar trend to that demonstrated between CD4 count recovery and progression to new AIDS diagnosis. The highest mortality rate occurred in the first six months beyond suppression (2.2/1000 PY; 95% CI: 1.6–2.8), and declined after that (6–12 months after suppression 1.9/1000 PY; 95% CI: 1.4–2.6 to 18–24 months post suppression 1.6/1000 PY; 95% CI: 1.1–2.4).

### Six-month CD4 recovery and progression to new AIDS condition

**Figure 1:**
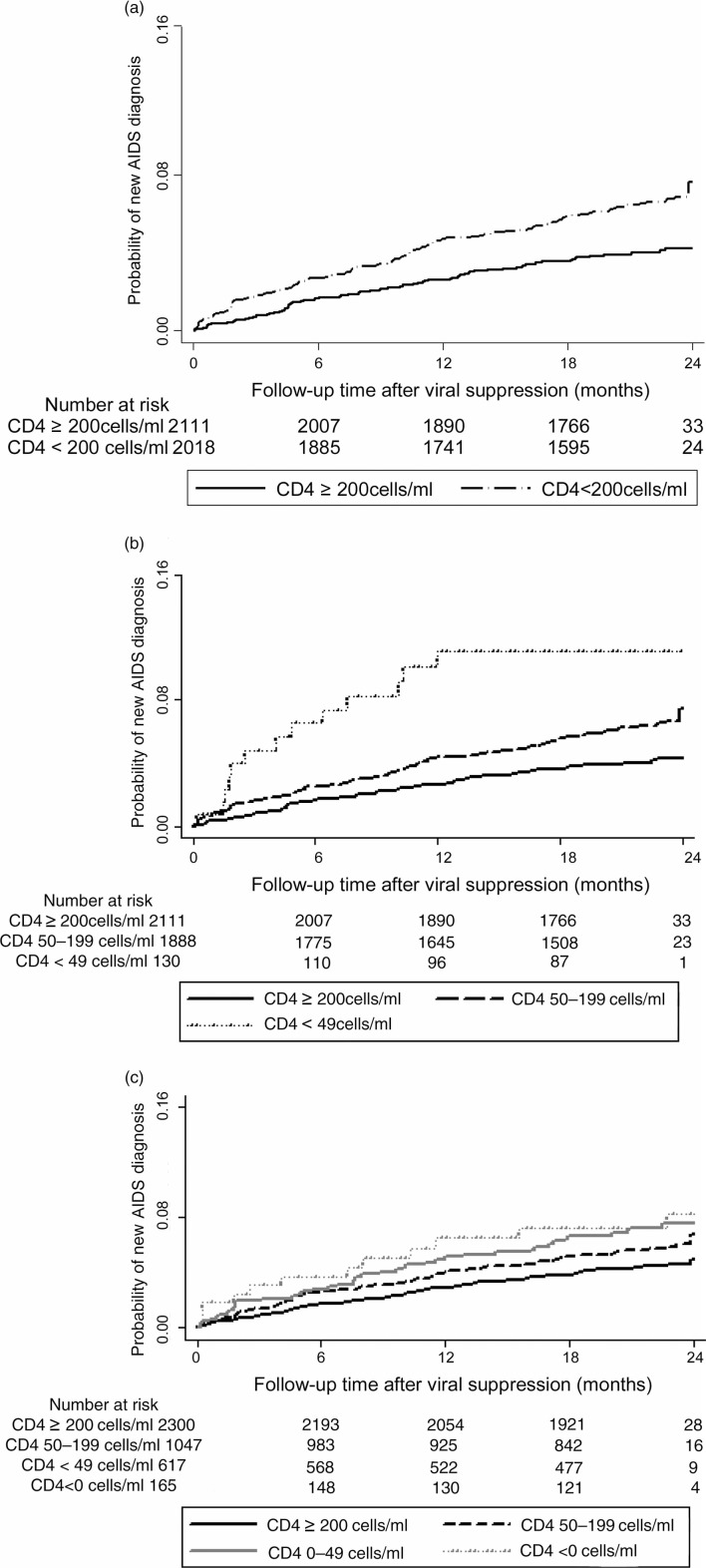
Kaplan–Meier Curves for progression to new AIDS condition by CD4 count response at viral suppression among 4129 patients initiating ART at the Themba Lethu Clinic in Johannesburg, South Africa.

In Kaplan–Meier analysis (Figure 1a, b, c), the risk of developing new AIDS condition was much higher among those with poor CD4 recovery regardless of the definition used.

In adjusted Cox regression models, an absolute CD4 count <200 cells/ml at six months post-ART initiation was associated with greater risk of progression to a new AIDS condition (aHR: 1.6; 95% CI: 1.2–2.2) versus ≥200 cells/ml (Table 2). However, when further divided, absolute CD4 counts <200 at six months were associated with approximately 2.0- to 3.0-fold increase in the risk of a new AIDS-defining condition, compared to a CD4 count ≥200 cells/ml. A decrease in CD4 count achieved by six months (aHR: 2.4; 95% CI: 1.2–4.9) and small gains (0–49 cells/ml; aHR: 2.0; 95% CI: 1.2–3.4 and 50–99 cells/ml; aHR: 1.5; 95% CI: 0.9–2.2) were associated with 1.5- to 2.4-fold risk with respect to risk of progression to AIDS when compared to a change of ≥200 cells/ml.

### Six-month CD4 count recovery and subsequent death

**Figure 2:**
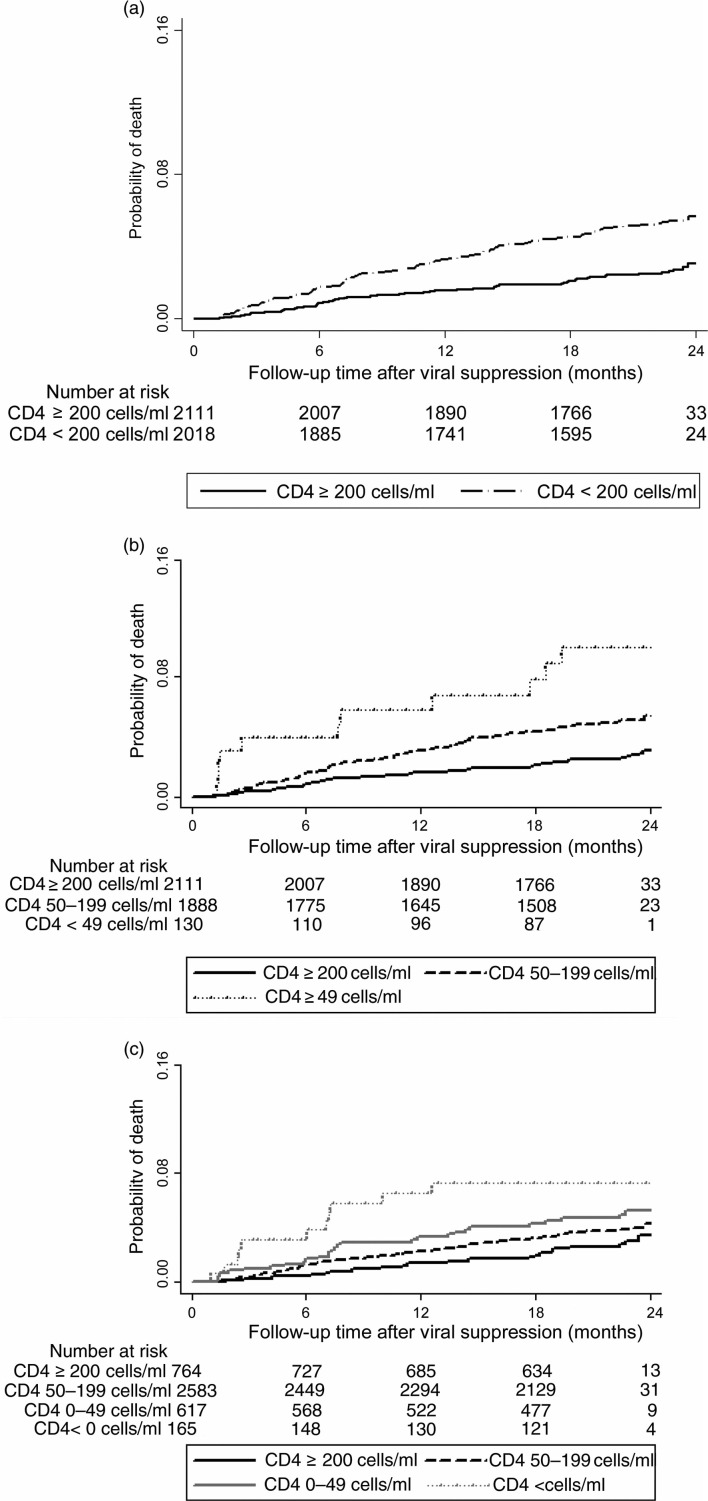
Kaplan–Meier Curves for progression to death by CD4 count response at viral suppression among 4129 patients initiating ART at the Themba Lethu Clinic in Johannesburg, South Africa.

In Kaplan–Meier analysis, excess mortality among those with poor CD4 recovery regardless of the definition used was observed. This is shown in Figure 2a, b, c.

In adjusted Cox regression model, the risk for death was again higher for patients failing to achieve an absolute CD4 count ≥200 cells/ml by six months on ART (aHR: 1.8, 95% CI: 1.3–2.6) versus ≥200 cells/ml (Table 2). A low absolute CD4 count at six months (0–49 cells/ml; aHR: 2.8; 95% CI: 1.4–5.7; and 50–200 cells/ml; aHR: 1.8; 95% CI: 1.3–2.6 was associated with an increased risk of death compared to CD4 count ≥200 cells/ml. A decrease in CD4 count (aHR: 2.0; 95% CI: 0.9–4.4) and smaller CD4 count gains (0–49 cells/ml; aHR: 1.5; 95% CI: 0.8–2.7 and 50–99 cells/ml; aHR: 1.3; 95% CI: 0.8–2.1) achieved by six months showed a trend towards greater risk of death when compared to a change of ≥200 cells/ml.

### Progression to new AIDS condition and death among virally suppressed patients by gender

The proportion of males and females progressing to new AIDS condition after viral suppression was not statistically different, 5.9% versus 4.7%, *p=*0.10 (males vs. females, respectively). In unadjusted Cox regression models, new AIDS condition rates were also not significantly different between males and females: HR: 1.27; 95% CI: 0.96–1.67 for males versus females (Table 3). In multivariable analyses adjusted for age, CD4 count, Hb, BMI and pregnancy status at six months post-ART initiation, event rates were also similar: aHR: 1.13; 95% CI: 0.84–1.52 for males versus females.

**Table 3:** Risk of progressing to new AIDS condition or death by gender at viral suppression among the 4129 patients initiating ART at the Themba Lethu Clinic in Johannesburg, South Africa

	Male	Female
New AIDS condition (*n=*212)		
Total, *n* (%)	87 (5.9%)	125 (4.7%)
Crude HR (95% CI) *p*	1.27 (0.96–1.67) 0.09	1.00 (ref)
Adjusted HR (95% CI) *p*	1.13 (0.84–1.52) 0.41	1.00 (ref)
Death (*n=*154)		
Total, *n* (%)	70 (4.7%)	84 (3.2%)
Crude HR (95% CI) *p*	1.52 (1.11–2.09) 0.01	1.00 (ref)
Adjusted HR (95% CI) *p*	1.39 (0.98–1.95) 0.06	1.00 (ref)

HR estimated using Cox proportional hazards regression models, adjusted for age, CD4 count, haemoglobin (Hb), body mass index (BMI) and pregnancy status at six months post-ART initiation.

When we examined mortality differences by gender among this virally suppressed cohort, a higher proportion of males died compared to females, 4.7% versus 3.2%, *p=*0.01. Higher hazards of death among males were also observed in unadjusted Cox regression models: HR: 1.52; 95% CI: 1.11–2.09. However, after adjustment, the estimates did not reach statistical significance: aHR: 1.39; 95% CI: 0.98–1.95 (Table 3).

## Discussion

In this South African cohort of over 4000 treatment naive patients initiating first-line ART, we show that despite achieving viral suppression by six months post-initiation, poor CD4 count recovery was associated with increased risk of progression to new AIDS-defining condition and death over 24 months after viral suppression (events measured 6–30 months on ART). This risk was greatest in the first 6 to 12 months after initiation of ART. These findings demonstrate that in addition to achieving viral suppression during ART, CD4 count recovery soon after ART initiation should be an important treatment goal. Regardless of viral suppression status, monitoring CD4 count recovery during this time period presents an early opportunity to identify patients at risk of poorer prognosis.

Though a majority of studies show evidence for an increased risk for progression to AIDS or death among those with poor immune recovery, there have been a few conflicting results. Amongst a multicentre cohort study in Germany, patients with poor immune recovery on ART seemed to remain at increased risk for AIDS despite suppressive ART [28]. Subsequent risk of death was also strongly associated with poor CD4 recovery in Zambia [27]. In the United Kingdom, Collaborative HIV Cohort Study, sub-optimal CD4 increases were associated with an increased risk of death but not with new AIDS events [26]. In contrast, in Eastern Europe in a retrospective cohort study of 446 patients, clinical events were not higher among patients with poor CD4 recovery compared to those with complete CD4 recovery [33]. Similarly, mortality and new opportunistic infection (OI) rates were low and similar between groups with sub-optimal and optimal CD4 recovery, respectively in a North American cohort study [30]. In addition, there were no significant differences in the rates of AIDS-related clinical events among patients with and without complete CD4 recovery in the setting of viral suppression, amongst a prospectively studied cohort in Kampala, Uganda [31].

To date, there is no consensus in current HIV guidelines on the definition of adequate CD4 response. To explore this, we tested varying specifications of CD4 recovery in this cohort to determine their impact on prognosis. First, we defined an adequate response as achieving an increase in CD4 count by six months on ART above a threshold of 200 cells/ml. An absolute CD4 count at six months of <200 cells/ml (Model 1) was the strongest predictor of progression to new AIDS condition and death. This finding supports current recommendations for continued provision of primary prophylaxis against OIs among patients with CD4 count <200 cells/ml including those who are virologically suppressed [40].

We observed a trend of higher hazards of death among patients with poorer CD4 responses. Insufficient numbers of deaths in this virally suppressed population may have resulted in some of the wide confidence intervals in these estimates. Our results on the impact of CD4 recovery on risk of death show a trend similar to findings reported previously [19,20,26,28,32]. In a prospective observational cohort of 404 HIV-positive patients in the United States, patients failing to achieve a CD4 count ≥50 cells/ml at six months despite viral suppression were two times more likely to develop OIs or death [19]. This was further replicated by Graber *et al*. where patients with virologic suppression who failed to achieve an increase in CD4 counts of at least 50 cells/ml at six months were three times more likely to develop an AIDS-defining illness or death than complete responders [20]. In contrast to our findings, a prospective Ugandan cohort demonstrated similar clinical outcomes in virally suppressed patients who had complete and incomplete CD4 count recovery [31]. This study used similar definitions of CD4 recovery to those we applied but had a much smaller sample size and fewer events than ours.

In this study, 67.9% of new AIDS conditions were due to tuberculosis (TB). Our data emphasize the burden of TB even among virally suppressed patients. Therefore, strategies that improve CD4 recovery could also reduce the TB-related morbidity and mortality. Lawn *et al*. demonstrated that reducing time patients spent with lower CD4 count reduced deaths from OIs [41]. This suggests it is important to continue serial CD4 count measurements in the first year post-ART initiation, even after achieving viral suppression. Appropriate prophylaxis for OIs hence should be continued in these patients. Though none of the patients were on any prophylaxis for TB (Isoniazid prophylaxis), all patients with CD4 count less than 200 cells/ml received cotrimoxazole prophylaxis. This raises the possibility of diminished effectiveness of prophylaxis in patients with poor CD4 count recovery such that even if this group of patients are on appropriate prophylaxis this may not be as effective as in patients with higher CD4 count and better CD4 count recovery. This needs further exploration. Our findings show high rates for TB as a new AIDS diagnosis among those with poor CD4 recovery. This is different to data observed in high-income settings where TB risk is low. Although we appreciate that the burden of TB is very high in our setting, the high rates of TB may be a reflection of the completeness of TB data versus other OI data due to increased vigilance over TB in the clinical setting and in addition, improved integration into HIV care of co-infected patients. It is unlikely that a significant proportion of new AIDS events measured could have been unmasking of pre-existing OI or pre-existing TB given that we measured outcomes at least six months after ART initiation. The immune reconstitution inflammatory syndrome (IRIS) occurs mostly soon after treatment initiation and in the SAPiT clinical trial, the median number of days from ART initiation to IRIS ranged from 17 to 28 days [42]. In addition, the patient's concomitant medications should be examined to exclude any that may be bone marrow suppressive, thereby suppressing CD4 production. The ART regimen should be continued because there is currently little data supporting switch or intensification to other regimens, though there is evidence that regimens containing ritonavir-boosted protease inhibitors, maraviroc and raltegravir may result in better CD4 recovery than EFV-based regimens [43–45]. This approach is being evaluated in controlled intervention studies. Interleukin-2 is an investigational agent that has been shown to increase CD4 cell counts substantially when given in addition to ART than ART alone, but studies evaluating this approach have not shown any translation to clinical benefit [46,47]. Intensifying ART adherence in such patients also needs to be explored. In order to maximally suppress viral load, studies show that one does not need to be 100% adherent; however, it is possible that some low level of circulating virus may continue to deplete CD4 cells [48].

Studies have shown inconsistent results regarding possible gender-differences disease progression and mortality among HIV-positive patients [49–55]. However, a large body of work in resource-limited settings points to increased mortality risk among males [54,55]. In a study conducted in the United States, comparing men and women at a similar stage of disease at presentation to care, women had higher mortality risk than men. However, the sex difference in baseline Hb levels (women had lower Hb levels) was postulated to significantly contribute to survival differences [53]. In a large study examining patients initiating ART between 2002 and 2009 at sites across South Africa, men had higher mortality than women. The authors hypothesized that gender differences in mortality could be explained by differences in baseline characteristics, lost-to-follow-up rates, and/or virologic and immunologic responses. In the present study, where all patients had achieved virologic suppression, we adjusted for important risk factors for mortality and disease progression that included age, CD4 count, Hb and BMI after the viral suppression. No significant differences were apparent except for a non-significant trend towards survival advantage among females. More studies investigating the role of gender among patients who have already achieved viral suppression are required.

The major strength of this study is the homogeneity of our study population. The study population was uniformly viral suppressed at baseline (<400 copies/ml) so that incomplete viral suppression would not explain our findings. In addition we also used routine programme data that commonly reflects limited resource populations. As commonly seen in resource-limited settings, this cohort was immunosuppressed at ART initiation (median CD4 count of 80 cells/ml), all were ART-naïve, and they began standard first-line ART (NNRTI based regimens). Our large sample size and large number of outcome events to draw from is a distinct advantage, in comparison to previous studies, which had lower sample size and fewer events. Our study findings, therefore, add to the body of knowledge of understanding the clinical relevance of poor CD4 responses in the setting of viral suppression.

Our findings also should be interpreted in light of the study limitations. First, the setting of this study is mainly an outpatient facility and this may result in underestimation of other serious clinical events, given that sicker patients are more likely to be admitted into hospital care. Second, because we only explored the relation between CD4 count at six months in our analysis, our data cannot be used to comment on the impact of long-term CD4 responses on poor clinical outcomes. The exclusion of patients (7.3%) with missing CD4 and viral load measurements at baseline and six months may have led to some selection bias. However, we expect this bias to be limited, because both excluded and included patients had mostly similar baseline characteristics. Use of cotrimoxazole prophylaxis may be an important confounder in this analysis. A recent systematic review and meta-analysis showed that data from low and middle-income countries did demonstrate that cotrimoxazole significantly increased survival in HIV-positive adults on ART [56]. We, however, did not have this data. As per South African national guidelines, all patients with CD4 count less than 200 cells/ml were expected to be started on cotrimoxazole prophylaxis and we are confident of compliance to this recommendation. Lastly, there was considerable lost to follow-up among the study subjects and this could have led to underestimation of the rates of new AIDS diagnosis and death.

In conclusion, our findings show the prognostic role of initial CD4 recovery during ART even among virally suppressed patients. Treatment success of ART should not be solely defined on viral suppression but interpreted as a combination of viral suppression and adequate CD4 response. Early CD4 count recovery regardless of viral suppression could be used as an additional tool used to identify patients who require intensified screening and diagnostic workup for OIs. Furthermore, early initiation of ART would be a major intervention to prevent CD4 count from getting too low and improving CD4 count recovery. Future studies need to focus on interventions that improve CD4 recovery among patients on ART.
